# Trends and Determinants in Breastfeeding among Korean Women: A Nationwide Population-Based Study

**DOI:** 10.3390/ijerph182413279

**Published:** 2021-12-16

**Authors:** Youn Huh, Yu Na Kim, Young Sik Kim

**Affiliations:** 1 Department of Family Medicine, Uijeongbu Eulji Medical Center, Eulji University, Uijeongbu-si 11749, Korea; higjdus1@naver.com; 2 Department of Family Medicine, Asan Medical Center, University of Ulsan College of Medicine, Seoul 05030, Korea; aladya1105@gmail.com

**Keywords:** breastfeeding, breastfeeding rate, smoking, determinants of breastfeeding, trend, Korea

## Abstract

Many efforts have been launched to promote breastfeeding in Korea. However, breastfeeding trends and associated factors with breastfeeding in Korea remain unknown. This study aimed to examine trends and determinants in breastfeeding using the Korea National Health and Nutrition Examination Survey (KNHANES) (2010–2018). We analyzed data from the KNHANES V (2010–2012), VI (2013–2015), and VII (2016–2018). A total of 9232 women aged 19–49 years were included in this study. We performed multivariable logistic regression analyses to investigate breastfeeding prevalence trends and associated factors with breastfeeding. Compared to 2010–2012, the odds ratio associated with breastfeeding during 2013–2015 and 2016–2018 increased to 1.30 (95% confidence interval (CI): 1.11–1.51) and 1.40 (95% CI: 1.21–1.63), respectively. The breastfeeding rate was associated with 40–49 years (OR, 95% CI: 0.47, 0.34–0.64 compared to 19–29 years), richer and poorer income (1.20, 1.03–1.39 in richer group and 1.24, 1.05–1.46 in poorer group compared to richest group), education level (0.74, 0.65–0.86 in ≤12 years of education compared to ≥13 years of education), smoking status (1.77, 1.38–2.28 in non-smoking compared to smoking), and self-rated health (1.40, 1.14–1.70 in good and 1.20, 1.00–1.44 in average compared to bad). Education programs and policies such as the Baby-Friendly Hospital Initiative (BFHI) and mother-friendly workplaces are necessary to increase the rates of breastfeeding in these groups.

## 1. Introduction

Breastfeeding is beneficial to both infants and mothers. It provides the nutrients that infants need for healthy development, reduces the morbidity of common childhood diseases such as diarrhea, asthma, and lower respiratory tract infections, and may improve cognitive development in children [1]. In addition, some studies have shown that early breastfeeding cessation increases the risk of early consumption of high-calorie beverages, increasing the risk of childhood obesity [2]. Breastfeeding has also been linked to the lower risk of maternal breast and ovarian cancer, obesity, and reduced medical costs [3]. However, despite these advantages, only 40% of infants aged 0–6 months received nutrition through breastfeeding [4].

The World Health Organization (WHO) recommends exclusive breastfeeding until 6 months of age and substituted breastfeeding up to 24 months of age. The World Health Assembly of the WHO in 2012 established a comprehensive plan for maternal and child nutrition to be achieved by 2025 [5]. Meanwhile, the Fourth Comprehensive Health Plan 2020 of the Ministry of Health and Welfare of the Republic of Korea aims to increase the breastfeeding rate of infants aged 0–6 months from 60.8% in 2010 to 66.8% in 2020 [6,7]. 

According to a breastfeeding survey conducted in Korea, the rate of breastfeeding was high in the 1980s and 1990s. Subsequently, due to factors such as industrialization, a lack of awareness of the importance of breastfeeding, and the absence of policies to support breastfeeding, the rate of breastfeeding decreased, reaching historically low levels in the 2000s. One study [1] showed that the breastfeeding rate at 1 month was 61.6% in 2001, 80.5% in 2005, and 86.0% from 2009 to 2011. In addition, the rate of exclusive breastfeeding is lower in Korea than other countries. The rate of exclusive breastfeeding was 9.4% at 6 months in Korea [8], instead of 22.3% at 6 months in the US [9]. For one of many efforts to promote breastfeeding, The WHO started the Baby-Friendly Hospital Initiative (BFHI) in 1991, and BFHI has grown over many countries worldwide, including Korea [10]. However, the number of BFHI-certificates awarded to hospitals is less than 20 nationally [10]. 

Factors associated with breastfeeding rate are education level [11], smoking status [12], and obesity [13]. However, studies on trends and factors associated with breastfeeding in Korea are limited. This study aimed to investigate associated factors and trends in breastfeeding for at least 1 month among women aged 19–49 years included in the National Health and Nutrition Examination Survey (KNHANES) (2010–2018).

## 2. Materials and Methods

### 2.1. Data Source and Study Participants

This cross-sectional study analyzed data from the V (2010–2012), VI (2013–2015), and VII (2016–2018) KNHANES. The KNHANES comprises a health interview such as smoking status, alcohol consumption, and past medical history and nutrition (including total calorie, fat, and protein intake and health examination surveys). It provides data on demographic characteristics, health behaviors, and health status collected through personal interviews, as well as data obtained from physical examinations along with blood sampling performed in mobile examination centers. A stratified, multistage probability sampling design was used to select the household units that participated in the survey.

**Figure 1:**
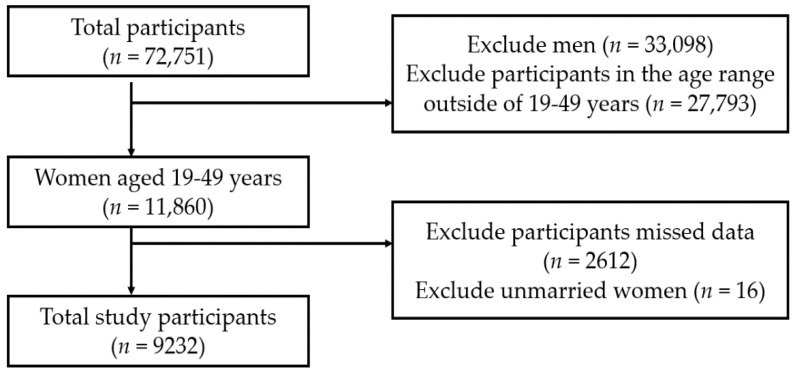
Study profile.

We included data on individuals who participated in the KNHANES from 2010 to 2018 (*n* = 72,751). We excluded men (*n* = 33,098), participants in the age range outside of 19–49 years (*n* = 27,793), those who had any missing variables (*n* = 2612), or those who were not married (*n* = 16). We excluded unmarried women because of selection bias. Finally, data of 9232 women were included in the analysis (Figure 1). Although the childbearing age was defined as 15–49 years in the Population Trend Survey of Statistics Korea, the target age for our study was 19–49 years because the age limit of childbirth and breastfeeding survey in the KNHANES was ≥19 years [14].

The survey was approved by the Institutional Review Board of Korea Centers for Disease Control and Prevention (IRB No: 2010-02CON-21-C, 2011-02CON-06-C, 2012-01EXP-01-2C, 2013-07CON-03-4C, 2013-12EXP-03-5C, and 2018-01-03-9-A). Our study adhered to the principles of the Declaration of Helsinki.

### 2.2. Definition of Any Breastfeeding

Participants that had given birth and answered “yes” to the question “Have you ever breastfed for at least 1 month” were defined as “any breastfeeding”. 

### 2.3. Measurements of Cardiometabolic Variables and Lifestyle Factors

Age was divided into three groups: 19–29, 30–39, and 40–49 years. Monthly household income level was categorized into quartile-based groups: the “poorest”, “poor”, “rich”, and “richest”. Educational level was divided into two groups: ≤12 and ≥13 years. Occupation was divided into three groups: “housewife”, “manufacturing/service”, and “office worker”.

People who had smoked at least 100 cigarettes in their lifetime and continued smoking at the time of the survey were defined as current smokers. Alcohol drinker was defined as a consumer of ≥1 drink per week, on average. Activity limitation was divided into two groups according to the presence or absence of activity limitation. Self-rated health status was categorized into three groups: “good”, “average”, and “poor”.

### 2.4. Statistical Analyses

We combined data from the 2010–2018 KNHANES based on the raw data analysis guidelines of the KNHANES. Moreover, based on the complex sample design, we conducted all analyses by assigning a dispersed stratification estimation, stratification variables, and weighted sample values according to the KNHANES statistical guideline. Continuous variables were analyzed in the general linear model and were presented as the mean and standard error. Categorical variables were presented as ratios and standard errors and were analyzed by the Chi-square test. A time-series analysis of breastfeeding was conducted based on data from the period of 2010–2012. Multivariable logistic regression analysis was performed to estimate the odds ratios (OR) of the periods 2013–2015 and 2016–2018 and their 95% confidence intervals (CI) to examine the associated factors and trend. A *p*-value of *<*0.05 was considered statistically significant. All analyses were performed using SPSS ver. 21.0 (IBM Corp., Armonk, NY, USA).

## 3. Results

### 3.1. Trends in the Prevalence of Breastfeeding among Women Aged 19–49 Years from 2010 to 2018

**Figure 2:**
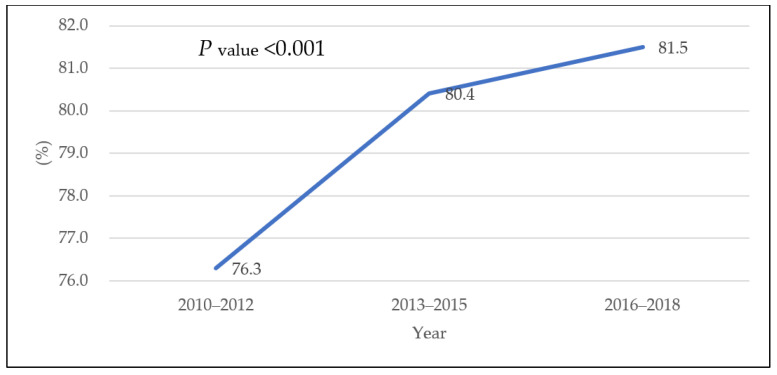
Trends in the prevalence of breastfeeding among women aged 19–49 years from 2010 to 2018.

Figure 2 and Table 1 show the changes in the breastfeeding rate among women aged 19–49 years from 2010–2012 to 2016–2018. The breastfeeding rate increased from 76.3% in 2010–2012 to 80.4% in 2013–2015 and 81.5% in 2016–2018 (*p* < 0.001). It increased among the age groups of 30–39 years and 40–49 years (*p* < 0.001, *p* = 0.004, respectively) but did not change in the age group of 19–29 years. In the richest income group, the rate of breastfeeding increased from 71.3% in 2010–2012 to 81.8% in 2016–2018 (*p* < 0.001); it also increased in the ≥13 years of education group (*p* = 0.001), among office workers (*p* = 0.002), among non-smokers (*p* < 0.001), and among non-drinkers (*p* = 0.005). Despite an initial increase, this rate decreased among binge drinkers (*p* = 0.001). Finally, the breastfeeding rate increased in the no activity restriction group (*p* < 0.001), and among participants in “good” self-rated health (*p* = 0.012); among the participant in “average” self-rated health, this rate initially increased, and then it decreased (*p* = 0.006).

### 3.2. Basic Characteristics of Women Aged 19–49 Years from 2010 to 2018

Table 2 presents the basic characteristics of the study participants (*n* = 9232). The prevalence of breastfeeding was highest among women aged 19–29 years (85.9%), followed by those aged 30–39 years (84.8%). The poorer group was associated with the highest prevalence of breastfeeding, followed by the richer and richest groups. The participants who received ≥13 years of education had a higher prevalence of breastfeeding than those who received ≤12 years of education. 

The breastfeeding rate was the highest among office workers (80.2%) and lowest among manufacturing/service workers. The breastfeeding rate was higher in the non-smoking group (80.0%) than in the smoking group and higher in the no-drinking group (80.2%) than in the drinking group. The breastfeeding rate was also higher among women without activity restrictions than among those with activity restrictions, and women with “good” self-rated health were more likely to breastfeed their babies (81.6%) than their counterparts. 

### 3.3. Factors Associated with Breastfeeding Rate in Women Aged 19–49 Years from 2010 to 2018 

Table 3 shows the factors associated with the breastfeeding rate among women aged 19–49 years from 2010 to 2018. The OR for breastfeeding increased to 1.30 (95% CI: 1.11–1.51) in 2013–2015 and 1.40 (95% CI: 1.21–1.63) in 2016–2018, compared to the 2010–2012 values. The OR for breastfeeding in 40–49 years decreased to 0.47 compared to 19–29 years (OR, 95% CI: 0.47, 0.34–0.64). Compared to the richest group, the ORs for breastfeeding increased to 1.20 and 1.24 in richer and poorer income, respectively (1.20, 1.03–1.39 in richer group and 1.24, 1.05–1.46 in poorer group). The breastfeeding in ≤12 years of education decreased to 0.74 compared to ≥13 years of education (0.74, 0.65–0.86). The OR for breastfeeding in non-smoking women increased to 1.77 compared to smoking women (1.77, 1.38–2.28). Compared to bad self-rated health, the ORs for breastfeeding increased to 1.20 and 1.24 in good and average self-rated health, respectively (1.40, 1.14–1.70 in good and 1.20, 1.00–1.44 in average). However, breastfeeding was not associated with age of 30–39 years, poorest group, occupation, alcohol consumption, or limitation of activity.

## 4. Discussion

This study examined trends in breastfeeding rates among Korean women in the past nine years using nationally representative data. The breastfeeding rate was 76.3% in 2010–2012, and it increased to 80.4% in 2016–2018 and 81.5% in 2016–2018. Breastfeeding was associated with age, income, education level, smoking status, and self-rated health.

According to the Ministry of Health, Labour, and Welfare in Japan, half of mothers continue exclusive breastfeeding with their children up to 3 months postpartum [15]. The ever-breastfeeding rate in Asia was 96.1% in China, 96% in Singapore, and 95.5% in India [16]. According to the Centers for Disease Control and Prevention, the percentage of breastfed babies continuously rose from 76.7% to 84.1% from 2010 to 2017 in the USA [17]. A 2015 European Federation of the Associations of Dietitians survey reported that 56%–98% of infants in Europe had been breastfed. By country, the ever-breastfeeding rate is 55% in Ireland, 95% in Norway, 86% in Italy, 98% in Sweden, 95% in Switzerland, and 92% in Finland [16]. The breastfeeding rate in Korea is slightly lower than that in the United States and is comparable to that in Europe. 

Income level influences breastfeeding, but this relationship is complex. A breastfeeding epidemiology study published in 2016 reported that high-income families breastfed more in high-income countries [18]. The breastfeeding rate significantly increased in the group with ≥13 years of education compared to the group with ≤12 years of education. This finding is consistent with that of the 2018 Korea Institute for Health and Social Affairs study on breastfeeding experiences of married women aged 15–49 years, where the breastfeeding rate tended to increase with increasing education level; both surveys covered a similar time period [19]. In addition, several studies have shown that education level is a more potent predictor of breastfeeding than other factors such as income level and mother’s occupation [20,21,22]. The breastfeeding rate and change thereof differed according to the type of occupation. The breastfeeding rate is lower among sales workers, service workers, and technicians than among professionals and managers [23]. Another study reported that the most effective strategies for maintaining breastfeeding after returning to work might include pumping during work hours, direct feeding, or a combination thereof; this study also suggested on-site childcare, teleworking, bringing the infant to work, and having the infant stay at the workplace as ways to support direct feeding [24].

The breastfeeding rate significantly increased among non-smokers compared to smokers. A 2016 meta-analysis showed that smoking is one of the most potent and consistent factors predicting early breastfeeding [25]. Furthermore, 50–80% of women who abstained from smoking during pregnancy resumed smoking within 6 months after delivery, which shortened the duration of breastfeeding and the amount of breast milk produced. Hence, abstinence from cigarette smoking during breastfeeding may help promote breastfeeding. Smoking is not a contraindication for breastfeeding. The reason that smoking women are less likely to breastfeed their babies than their non-smoking counterparts involves the physiological impact of smoking on breast milk production and lower motivation for breastfeeding [26]. According to a report on the changing trends in health behavior and chronic disease by the Korea Disease Control and Prevention Agency, the smoking rate among women aged 20–49 years has risen since 2001 and remains approximately twofold higher than that recorded 20 years ago [27]. 

The breastfeeding rate was associated with self-rated health. Overweight and obese women with labor-related complications or medical problems are less likely to initiate breastfeeding, and obese women without any medical problems are at an 11% higher risk of stopping breastfeeding than their non-obese counterparts [28]. Thus, breastfeeding women should be assessed for factors such as obesity, medical history, temporary complications, and drinking and smoking status. 

Several exercise programs and projects have been launched to promote breastfeeding, including the Baby-Friendly Hospital Initiative and Mother-Friendly Workplace projects, which provide breastfeeding education using videos and other materials [29]. The breastfeeding rate increases with increasing exposure to baby-friendly hospital projects, including rooming-in; postpartum care centers in Korea emphasize the importance of rooming-in, with a growing number of baby-friendly hospitals [30,31]. Further, the Mother-Friendly Workplace project aims to implement facilities and systems that support breastfeeding even after returning to the workplace. There are 15 baby-friendly hospitals and 33 mother-friendly workplaces in Korea as of March 2021 [31]. Various programs and projects are needed to increase the breastfeeding rate in Korean women.

## 5. Limitations and Strengths

This study has some limitations. First, as participants’ health status, lifestyle, and breastfeeding information was self-reported, the data could be subject to recall bias. Second, this study used the KNHANES data, and the rates of breastfeeding and exclusive breastfeeding/combination feeding within a particular period were not surveyed. Third, due to the nature of cross-sectional studies, the external validity of our study is poor. Finally, while we adjusted for potential confounders, we could not consider variables not included in the KNHANES. 

Despite these limitations, this study has identified some factors that contribute to breastfeeding rates among contemporary women of childbearing age living in Korea. 

## 6. Conclusions

In conclusion, the breastfeeding rate in Korea has been increasing since 2010–2012 and remains low among Korean women compared to breastfeeding rates in India, China, the United States, and Europe. Breastfeeding was associated with age, income, education level, smoking status, and self-rated health. Hence, education programs and policies such as BFHI and mother-friendly workplaces targeting women are required. 

## Figures and Tables

**Table 1:** Trends in the prevalence of breastfeeding among women aged 19–49 years from 2010 to 2018 in subgroups.

Classification	Variables	2010–2012 (*n* = 3475)	2013–2015 (*n* = 2792)	2016–2018 (*n* = 2965)	*p*-Value
		% (SE)	% (SE)	% (SE)	
Age (years)	19–29	86.8 (2.8)	87.3 (3.1)	82.3 (3.5)	0.528
	30–39	80.9 (1.4)	86.0 (1.3)	88.3 (1.0)	<0.001
	40–49	71.0 (1.3)	75.2 (1.3)	76.7 (1.2)	0.004
Income	Richest	73.9 (3.4)	75.7 (4.3)	81.2 (2.8)	0.302
	Richer	77.7 (1.7)	80.7 (1.6)	83.2 (1.6)	0.058
	Poorer	79.9 (1.4)	82.2 (1.4)	80.0 (1.5)	0.436
	Poorest	71.3 (1.7)	79.1 (1.6)	81.8 (1.3)	<0.001
Education level	≤12 years	74.4 (1.1)	77.2 (1.3)	76.5 (1.4)	0.230
	≥13 years	79.1 (1.3)	84.0 (1.2)	85.2 (0.9)	0.001
Occupation	Housewife	77.8 (1.2)	81.5 (1.3)	81.4 (1.3)	0.058
	Manufacturing/service	74.6 (1.8)	77.9 (1.7)	79.8 (1.7)	0.077
	Office worker	75.3 (1.9)	81.1 (1.5)	82.9 (1.3)	0.002
Alcohol consumption	(−)	77.9 (1.2)	80.2 (1.3)	83.6 (1.2)	0.005
	(+)	74.5 (1.3)	80.7 (1.2)	79.8 (1.2)	0.001
Smoking status	(−)	76.9 (0.9)	80.9 (0.9)	82.5 (0.8)	<0.001
	(+)	66.6 (4.5)	72.5 (4.2)	66.8 (4.1)	0.548
Limitation of activity	(+)	73.2 (4.1)	76.2 (4.7)	69.3 (5.1)	0.637
	(−)	76.4 (0.9)	80.6 (0.9)	81.8 (0.8)	<0.001
Self-rated health	Good	78.6 (1.6)	82.5 (1.3)	84.4 (1.3)	0.012
	Average	76.0 (1.3)	80.8 (1.2)	80.6 (1.1)	0.006
	Bad	71.3 (2.8)	74.2 (2.7)	78.0 (2.2)	0.190

Abbreviations: SE, standard error. Data are presented as percentages (SE). *p*-Values were obtained by using chi-squared test.

**Table 2:** Basic characteristics of women aged 19–49 years from 2010 to 2018.

Classification	Variables	Total	Breast Feeding (+)	
		(*n* = 9232)	(*n* = 7379)	% (SE)
Age (years)	19–29	443	386	85.9 (1.9)
	30–39	3940	3356	84.8 (0.7)
	40–49	4849	3637	74.3 (0.7)
Income	Richest	563	439	76.7 (2.1)
	Richer	2410	1949	80.2 (1.0)
	Poorer	3191	2590	80.7 (0.8)
	Poorest	3068	2401	77.4 (0.9)
Education level	≤12years	4544	3481	75.8 (0.7)
	≥13years	4688	3898	83.0 (0.7)
Occupation	Housewife	4276	3439	80.1 (0.7)
	Manufacturing/service	2385	1869	77.1 (1.0)
	Office worker	2571	2071	80.2 (0.9)
Alcohol consumption	(−)	8734	7024	80.0 (0.5)
	(+)	498	355	68.5 (2.5)
Smoking status	(−)	4551	3664	80.2 (0.7)
	(+)	4681	3715	78.3 (0.7)
Limitation of activity	(+)	342	255	73.3 (2.9)
	(−)	8890	7124	79.5 (0.5)
Self-rated health	Good	3164	2596	81.6 (0.8)
	Average	4842	3858	79.1 (0.7)
	Bad	1226	925	74.3 (1.5)

Abbreviations: SE, standard error. Data are presented as percentages (SE).

**Table 3:** Factors associated with breastfeeding rate in women aged 19–49 years from 2010 to 2018.

Classification	Variables	OR (95% CI)	*p*-Value
Period	2010–2012	1	
	2013–2015	1.30 (1.11–1.51)	0.001
	2016–2018	1.40 (1.21–1.63)	<0.001
Age(years)	19–29	1	
	30–39	0.86 (0.62–1.20)	0.375
	40–49	0.47 (0.34–0.64)	<0.001
Income	Richest	1	
	Richer	1.20 (1.03–1.39)	0.018
	Poorer	1.24 (1.05–1.46)	0.012
	Poorest	1.19 (0.92–1.54)	0.184
Education level	≤ 12years	0.74 (0.65–0.86)	<0.001
	≥ 13years	1	
Occupation	Housewife	1.02 (0.88–1.17)	0.816
	Manufacturing/service	1.13 (0.95–1.35)	0.171
	Office worker	1	
Alcohol consumption	(−)	1.12 (0.99–1.27)	0.067
	(+)	1	
Smoking status	(−)	1.77 (1.38–2.28)	<0.001
	(+)	1	
Limitation of activity	(+)	0.93 (0.69–1.27)	0.658
	(−)	1	
Self-rated Health	Good	1.40 (1.14–1.70)	0.001
	Average	1.20 (1.00–1.44)	0.048
	Bad	1	

Abbreviations: OR, odds ratio; CI, confidence interval. Values were calculated by multivariable logistic regression analysis, which was adjusted for period, age, income, education level, occupation, alcohol consumption, smoking status, limitation of activity, and self-related health.

## Data Availability

All data underlying the authors’ findings in this study are freely available in Korea Centers for Disease Control and Prevention. If interested in requesting these data, please visit the following link for more information: https://knhanes.cdc.go.kr/knhanes/main.do (accessed on 20 March 2021).
